# Feasibility Study of Mesoporous Silica Particles for Pulmonary Drug Delivery: Therapeutic Treatment with Dexamethasone in a Mouse Model of Airway Inflammation

**DOI:** 10.3390/pharmaceutics11040149

**Published:** 2019-04-01

**Authors:** Tina Gulin-Sarfraz, Sofia Jonasson, Elisabeth Wigenstam, Eva von Haartman, Anders Bucht, Jessica M. Rosenholm

**Affiliations:** 1 Pharmaceutical Sciences Laboratory, Faculty of Science and Engineering, Åbo Akademi University, 20520 Turku, Finland; tgulin@abo.fi (T.G.-S.); ehaartma@abo.fi (E.v.H.); 2 School of Pharmacy, University of Oslo, 0371 Oslo, Norway; 3 CBRN Defence and Security, Swedish Defence Research Agency, 90182 Umeå, Sweden; sofia.jonasson@foi.se (S.J.); elisabeth.wigenstam@foi.se (E.W.); 4 Department of Public Health and Clinical Medicine, Unit of Respiratory Medicine, Umeå University, 90182 Umeå, Sweden

**Keywords:** mesoporous silica particles, pulmonary drug delivery, poorly soluble drugs

## Abstract

Diseases in the respiratory tract rank among the leading causes of death in the world, and thus novel and optimized treatments are needed. The lungs offer a large surface for drug absorption, and the inhalation of aerosolized drugs are a well-established therapeutic modality for local treatment of lung conditions. Nanoparticle-based drug delivery platforms are gaining importance for use through the pulmonary route. By using porous carrier matrices, higher doses of especially poorly soluble drugs can be administered locally, reducing their side effects and improving their biodistribution. In this study, the feasibility of mesoporous silica particles (MSPs) as carriers for anti-inflammatory drugs in the treatment of airway inflammation was investigated. Two different sizes of particles on the micron and nanoscale (1 µm and 200 nm) were produced, and were loaded with dexamethasone (DEX) to a loading degree of 1:1 DEX:MSP. These particles were further surface-functionalized with a polyethylene glycol–polyethylene imine (PEG–PEI) copolymer for optimal aqueous dispersibility. The drug-loaded particles were administered as an aerosol, through inhalation to two different mice models of neutrophil-induced (by melphalan or lipopolysaccharide) airway inflammation. The mice received treatment with either DEX-loaded MSPs or, as controls, empty MSPs or DEX only; and were evaluated for treatment effects 24 h after exposure. The results show that the MEL-induced airway inflammation could be treated by the DEX-loaded MSPs to the same extent as free DEX. Interestingly, in the case of LPS-induced inflammation, even the empty MSPs significantly down-modulated the inflammatory response. This study highlights the potential of MSPs as drug carriers for the treatment of diseases in the airways.

## 1. Introduction

The respiratory tract is an attractive route for non-invasive drug delivery for the local treatment of lung diseases, such as asthma and cystic fibrosis. Specific lung cells can further be targeted for treating diseases such as tuberculosis and possibly even lung cancer [1,2,3]. The fact that respiratory diseases rank among the top ten causes of death globally (four out of ten) [4], makes this field of study highly important. In addition, as a result of the potential uptake of drugs across the respiratory epithelium, drug delivery through inhalation is also gaining importance for non-respiratory diseases, where the lungs are only considered for their potential as a portal of entry for pharmaceutically active compounds [5]. The respiratory tract offers several advantages over conventional oral drug delivery [6,7]. These include the highly vascularized and large surface area of the alveoli, which enables access to the microvascular circulation of the lungs, resulting in a faster drug adsorption, and also a possible circumvention of the first pass effect [8]. The effectiveness of the drug uptake is related to the amount of drug deposited in and the distribution within the airways [9].

Nanoparticles offer many unique advantages as therapeutic tools because of their design flexibility. In particular, silica nanoparticles have attracted much attention as being one of the most biocompatible drug carriers [10,11], as amorphous silica has also been classified as a “generally recognized as safe” (GRAS) material by the food and drug administration (FDA). To date, drug delivery systems based on silica nanoparticles have primarily been studied through intravenous administration, but recently gained importance for use also through the pulmonary route [12]. Mesoporous silica nanoparticles are bioerodible and have a high surface area that makes it possible to incorporate drug amounts equaling the particle’s own weight (1:1 drug:carrier) [13]. Silica is a highly flexible platform that can be specifically designed, depending on the desired application and administration route [14]. When particles enter the lungs, particle deposition occurs in the whole respiratory tract by different mechanisms. Particle size and geometry are the most important parameters for the deposition, next to the morphological characteristics and ventilation parameters of the lungs [15]. Thus, the ease of particle size control, size uniformity, and flexible surface functionalization possibilities make silica nanoparticles ideal as pulmonary drug carriers. Nanoparticle-based carriers can improve the pharmacokinetic and pharmacodynamic profiles of conventional drugs, and may thus optimize the efficacy of the drug [16]. Silica nanoparticles are hydrophilic, which is essential for any in vivo application, and the dispersibility of the particles in aqueous media can further be enhanced by the right surface functionalization [17]. Consequently, mesoporous silica particles have recently been highlighted as ideal carriers for poorly water-soluble drugs [18,19,20], which, despite a high potency, often have severely limited efficacy as a result of the fact that the amount of soluble drug is not high enough for reaching therapeutic concentrations.

Here, the possibility/feasibility of using mesoporous silica particles (MSPs) as carriers for anti-inflammatory drugs in the treatment of airway inflammation was investigated. Corticosteroids, such as dexamethasone (DEX), used in this study, are well-known to decrease the number of inflammatory cells in the airways, and to improve the respiratory function. However, DEX can also cause unwanted side effects, particularly when used at high doses [21,22,23,24]. As DEX is practically insoluble in water (0.1 mg/mL) [25], it would be expected that a silica particle-formulation can enhance the solubility and dissolution rate of the drug. Drug-loaded particles, compared to free drugs, also have a higher ability to reach the lower parts of the lungs [26,27]. DEX has potent anti-inflammatory properties and is an ideal therapeutic agent for acute airway inflammation. By being able to load particles with DEX in order to improve airway distribution and to enable local treatment, side effects may be reduced, and it may also be possible to use higher doses locally in the lung. In our study, particle sizes of 200 nm and 1 µm were synthesized for comparison, and loading degrees exceeding 100 wt % (1:1 drug:carrier) of DEX were obtained. The particles were further coated with polyethylene glycol—polyethylene imine (PEG–PEI) copolymer to improve the dispersibility of the drug-loaded MSPs, and to potentially promote favorable and to suppress unfavorable interactions upon administration via the airways.

Two different mice models of airway inflammation were utilized, as follows: (1) chemical-induced airway inflammation provoked by exposure to the cytotoxic compound melphalan (MEL), and (2) endotoxin-induced pulmonary inflammation caused by exposure to lipopolysaccharide (LPS). Previous studies have shown that MEL is a possible surrogate for the chemical warfare agent sulphur mustard, causing a neutrophilic airway inflammation in the acute phase (1–2 days post exposure) [24,28,29]. Inhalation of LPS induces an acute inflammation and has been studied extensively. One role of LPS is the activation and migration of blood leukocytes into the airways, the dominating type being neutrophils [30]. The two models have in common that there is a rapid inflammatory process in the acute phase, which peaks within 24 h. In both models, we have earlier demonstrated that therapeutic treatment with DEX 1 h (intraperitoneal injection) after exposure is effective for the prevention of the acute inflammation [24,31]. In this study, animals were treated with aerosolized free DEX or MSPs with and without loaded DEX, 1 h after exposure to MEL or LPS. Mice were evaluated for treatment effects 24 h after exposure; whereby the inflammatory cells and pro-inflammatory mediators (keratinocyte chemoattractant (KC), Matrix metalloproteinase-9 (MMP-9), and Mouse Myeloperoxidase (MPO)) were analyzed in bronchoalveolar lavage fluid (BALF).

## 2. Materials and Methods

### 2.1. Preparation of the Large (L-MSP) and Small (S-MSP) Mesoporous Silica Particles

The L-MSP and S-MSP were synthesized according to a procedure reported by D. Kumar et al. [32], with slight modifications. Briefly, 2 g hexadecylamine (Sigma-Aldrich, Merck KGaA, Darmstadt, Germany) was dissolved in 200 mL isopropanol, 180 mL milli-Q water, and 2.4 mL NH_3_ (33 wt %). For the synthesis of L-MSP, 11.6 mL tetraethyl orthosilicate (TEOS) was added to the solution as a silica source, while a mixture of 11 mL TEOS and 0.6 mL aminopropyl triethoxysilane (APTES) was added to the synthesis solution of S-MSP to reduce the resulting particle size. Both of the synthesis solutions were left for overnight reaction under stirring, whereafter the particles were separated by centrifugation (3360 g), and the surfactant template was removed by extraction two times for 1 h in slightly acidic ethanol (0.1 M). During extraction, the solutions were stirred and shaken, with no sonication involved, so as to preserve the porous structure of the particles. Finally, the particles were washed with ethanol and were vacuum-dried at room temperature (RT).

### 2.2. Loading of Drug to the Particles

20 mg of dexamethasone (DEX) was dissolved in 9 mL of anhydrous cyclohexane (Sigma-Aldrich, Merck KGaA, Darmstadt, Germany) by sonication. Then, 20 mg of vacuum-dried L-MSP or S-MSP were added, and the drug-particle suspension was ultrasonicated and vortexed repeatedly three times. The suspension was left under stirring overnight. The drug-loaded particles were separated by centrifugation (630 g), washed by cyclohexane, and vacuum-dried at RT.

### 2.3. Copolymer-Adsorption on the Particles

The particles were coated with an earlier developed PEG–PEI copolymer (mPEG_low_–PEI) [33] as follows. 10 mg of drug-loaded particles were dispersed in 1 mL of HEPES (4-(2-hydroxyethyl)piperazine-1-ethanesulfonic acid) buffer (25 mM, pH 7.2) by careful sonication so as to not leach out the drug. For the same reason, the particle-concentration was kept high. Then, 10 mg of the PEG–PEI copolymer was dissolved in 1 mL HEPES, and was added to the particle-suspension during sonication. The reaction was left for 3 h under stirring. The copolymer-coated particles were separated by centrifugation (3629 g), washed with a small amount of milli-Q water, and then vacuum-dried at RT.

Empty particles (without drug) were also coated with copolymer to later serve as the control particles in the in vivo studies.

### 2.4. Determination of the Loaded DEX Amount

The amount of DEX inside the pores of the PEG–PEI-coated particles was determined by elution of the drug in methanol. A particle concentration of 0.1 mg/mL in the methanol was repeatedly sonicated and vortexed for 1 h. The particles were centrifuged, and the supernatant was measured on a Ultraviolet–visible (UV-Vis) Spectrophotometer (NanoDrop 2000c, Thermo Fisher Scientific Inc., Waltham, MA, USA) at a wavelength of 242 nm. The amount of DEX was calculated by using a standard curve of DEX in methanol.

### 2.5. Animal Models

Female C57BL/6OlaHsd mice (9–10 weeks old) obtained from Envigo RMS B.V, Netherlands, were used in this study. The animals were fed with standard chow and water ad libitum. The care of the animals and the experimental protocols were approved by the regional ethics committee on animal experiments in Umeå, Sweden (A69-15, 20/11/15).

Two different mice models for airway inflammation were utilized, as follows:

1. Melphalan (MEL)-induced airway inflammation: The mice were briefly anaesthetized with isofluran and melphalan (4-(bis(2-chlorethyl)amino)-l-phenylalanine) (Sigma-Aldrich, St Louis, MO, USA) administered by intratracheal instillation in a volume of 50 µL (1 mg/kg). Melphalan was dissolved in acidic ethanol (30 µL concentrated HCl in 1 mL 99.5% ethanol) to a concentration of 100 mg/mL, and further diluted in phosphate buffered saline (PBS) to a final concentration just before administration [24]. The control mice received only the solvent.
2. Lipopolysaccharide (LPS)-induced airway inflammation: The mice were exposed to an aerosol of Lipopolysaccharide (LPS; *Escherichia coli* O128:B12; Sigma-Aldrich, Merck KGaA, Darmstadt, Germany) for 15 min using a nose-only Battelle exposure chamber. The aerosol was generated by a compressed-air Collison six-jet nebulizer at an airflow of 7 liters/min using a nebulizer concentration of 0.1 mg/mL of LPS dissolved in water [31]. The control mice were exposed to an aerosol of solvent alone.

#### 2.5.1. Treatment

The samples with MSPs with and without DEX were placed in an ultrasonic bath for about 15 min before administration and were shaken every 5 min. All of the samples were diluted in a HEPES buffer to a volume of 200 µL, and were administered as an aerosol using Aeroneb™ PRO, SCIREQ^®^. The mice were randomly allocated into different groups (listed in Table 1, *n* = 6 animals/group) and were placed in individual nose-only containers and via a connecting tube exposed to aerosolized droplets (diameter of 4–6 µm) of MSPs with and without DEX in various concentrations and sizes 1 h after the exposure to MEL or LPS. One group of animals were administered free DEX (5 mg/mL in HEPES buffer) without MSPs as a positive control group.

After 24 h post exposure, the animals were tracheostomized and the BALF was collected. The lungs were lavaged four times via a tracheal tube, with a total volume of 1 mL + 3 × 1 mL Ca^2+^/Mg^2+^ free ice cold Hank’s Balanced Salt Solution (HBSS; Sigma-Aldrich, Merck KGaA, Darmstadt, Germany). The BALF was centrifuged (524 g, 10 min, and 4 °C) and the supernatant was stored at −80 °C for later analysis. The cells in the pellets were dissolved in PBS and the number of leukocytes were counted in a Bürker chamber using trypan blue staining. There were 20,000 cells per animal that were fixed on duplicate slides using a Cytospin^®^ centrifuge (Shandon© cytospin 3 cyto-centrifuge, cell preparation system, Runcorn, UK) and were stained with May–Grünwald–Giemsa reagents (Merck Millipore, VWR International, Spånga, Sweden) before a differential count was performed in a blinded manner, counting 200 cells per slide. The total cells in the BALF and the percentage of neutrophils are presented in this study.

The BALF analysis was performed using ELISA kits, as follows: (1) Mouse CXCL1/KC DuoSet ELISA, (2) Mouse Myeloperoxidase (MPO) DuoSet ELISA, and (3) Matrix Metalloproteinase-9 (MMP-9; Mouse Total MMP-9 DuoSet ELISA), according to the manufacturer’s instructions (R&D systems™, Abingdon, UK), and were analyzed using an ELISA reader (Thermo Scientific Mutilskan FC, Thermo Fischer Scientific Oy, Vantaa, Finland). The analysis of the ELISA data was performed using the software program for the ELISA reader (SkanIt for Multiskan FC 3.1. Inc., Thermo Fischer Scientific Oy, Vantaa, Finland).

#### 2.5.2. Statistical Analysis of the Animal Data

The results are presented as the means ± standard error of means (SEM). The statistical significance was assessed by parametric methods using a one-way analysis of variance (ANOVA) to determine the differences between the groups, followed by a Bonferroni post hoc test. A statistical result of *p* < 0.05 was considered significant. The statistical analyses were carried out and graphs were prepared using the GraphPad Prism program (version 6.0 GraphPad software Inc., San Diego, CA, USA).

## 3. Results and Discussion

### 3.1. Design and Characterization of the Carrier Particles for the Anti-Inflammatory Drug

**Figure 1:**
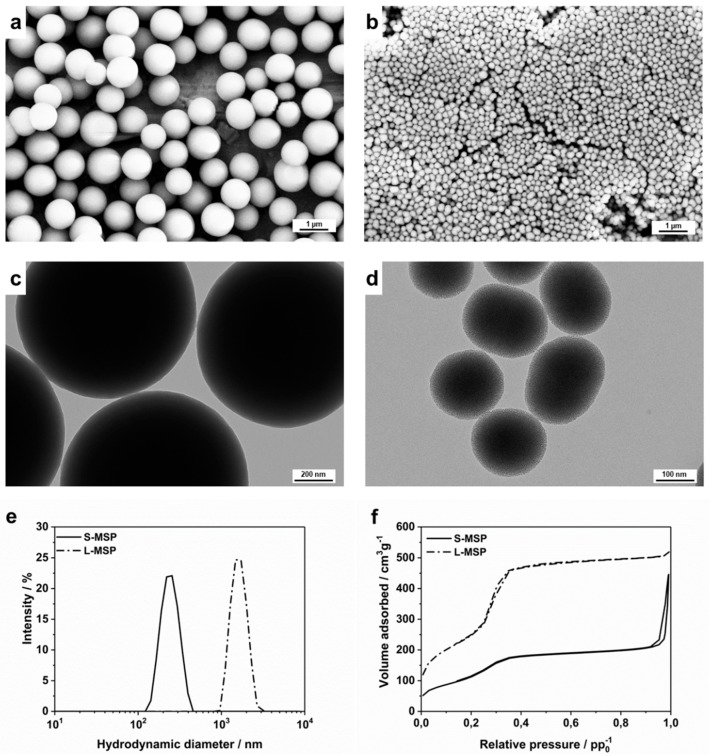
Characterization of the large mesoporous silica nanoparticle (L-MSP) and small (S-MSP) particles. (**a**) Scanning electron microscopy (SEM) and (**c**) transmission electron microscopy (TEM) images of the L-MSP. (**b**) SEM and (**d**) TEM images of the S-MSP. (**e**) Hydrodynamic size distributions of the particles. The peak for the S-MSP is centered at 230 nm with a PdI of 0.03, while the peak for the L-MSP is at 1500 nm with a PdI of 0.05. (**f**) Nitrogen sorption isotherms of the particles. The S-MSP has a BET surface area of 420 m^2^/g and a pore volume of 0.45 cm^3^/g, and the L-MSP has a BET surface area of 900 m^2^/g and a pore volume to 0.75 cm^3^/g.

The treatment effect of the DEX-loaded MSPs on two differently induced lung inflammation models was investigated by comparing two sizes of particles. Both the larger (L-MSP) and the smaller (S-MSP) particles were produced, with modifications from the synthesis reported in Kumar et al. [32], which yielded highly porous, spherical particles composed of a random close-packing of smaller primary nanoparticles. Electron microscopy images, as shown in Figure 1a–d, indeed reveal spherical and mono-dispersed particles, with the larger particles sized around 1 µm and the smaller less than 200 nm. The hydrodynamic sizes (Figure 1d) of the particles were 1.5 µm and 230 nm, respectively. Nitrogen sorption measurements (Figure 1f) confirm the mesoporosity of the particles. The specific surface area (Brunauer-Emmet-Teller, BET) was determined to be 900 m^2^/g for the L-MSPs, and the pore volume was 0.75 cm^3^/g. The S-MSPs had a BET surface of 420 m^2^/g, and a pore volume of 0.45 cm^3^/g. The average pore width was 3.7 nm for the L-MSP and 3.5 nm for the S-MSP.

Porous silica particles were obtained by adding a structure-directing agent (SDA), i.e., surfactants or polymers, to the synthesis solution; in this case hexadecylamine. The method for extracting the SDA from the as-synthesized particles has to be carefully chosen in order to keep the porous structure intact and to not cause aggregation/agglomeration of the particles, concomitant to properly removing all of the SDA. Here, we compared five different solvent extraction methods for the removal of the hexadecylamine surfactant template, which are explained more in detail in the Supplementary Information (Figures S1, S2, and S3). Briefly, an acidic solvent in combination with harsh treatment by sonication destroyed the morphology of the particles and caused aggregation, as can clearly be seen in Figure S1. Acidic solvent extraction with only stirring and/or shaking of the particle suspension did not damage the particles; nor did extraction by only isopropyl alcohol together with sonication. However, the nitrogen sorption measurements revealed a clear difference in the specific surface area and the pore volume of the particles extracted by acidic solvent compared to only alcohol (Figure S2), which suggest that alcohol alone cannot efficiently extract the SDA. The thermogravimetric measurements confirmed that some surfactant remained in the pores when only alcohol was used for the extraction (Figure S3). Thus, a careful extraction (without sonication) of the SDA by acidic solvent was used for further studies. This demonstrates the importance of using a method for the removal of the SDA, which matches the particle synthesis procedure and the chosen SDA. Many surfactants are highly toxic and even small residues of such substances can be very harmful.

Porous silica particles are well known for their capability to carry a large amount of drugs [18,19,20]. Here, the L-MSP and S-MSP were loaded with 100 wt % (drug/silica) DEX to in vivo ensure a high amount of drug in the inhaled dose. To further keep the drug-loaded particles properly dispersible for aerosol inhalation, a PEG–PEI copolymer developed in our previous study [33] was utilized for surface coating. The surface modification of particles, intended for use in vivo, is essential in order to keep the particles hydrophilic and to prevent aggregation, and consequently increase the biocompatibility. The copolymer was prepared from PEG (5 kDa) and PEI (25 kDa) at a grafting ratio of 2, which was shown in our previous study to be the ideal grafting ratio for optimal dispersibility [33]. The amount of DEX in the particles after surface functionalization was measured by the elution of DEX from the particles in methanol. DEX has a high solubility in methanol and is rapidly eluted from the silica matrix. The amount of drug in the supernatant could thus be calculated through the UV-Vis measurement of the supernatant, and reached about 100 wt % for both L-MSP and S-MSP, which shows that the drug was still retained in the silica matrix during the PEG–PEI coating.

When formulating poorly water-soluble drugs with the aim of dissolution enhancement, a rapid release of the drug from the carrier particles is a crucial requirement, and DEX has low solubility in aqueous solutions. However, when formulated as a molecular dispersion adsorbed into a silica matrix, an almost immediate release from the silica particles occurred. This was studied in vitro in HEPES buffer and was measured by UV-Vis spectrophotometry. The release kinetics over time are presented in Figure S4, and reveal a 50%–60% release after only 15 min, with an increase to around 90% after 3 h. This is in line with the report by Martín et al. [34], who studied the loading and release of the steroid drug prednisolone from different mesoporous silica carriers, and found that all of them released 70%–90% of the drug in 30 min, and the overall tendency of all of the silica particles studied indicated that more than 85% of the adsorbed drug was released in 5 h, independently of the material.

### 3.2. In Vivo Investigation of the Anti-Inflammatory Response Induced by Drug-Loaded MSPs

The drug-loaded L-MSPs and S-MSPs were further studied in vivo in two different mice models of airway inflammation. In one group, the airway inflammation was induced by melphalan (MEL) and in the other group by lipopolysaccharide (LPS), 1 h prior to the study. A nebulizer was utilized for the administration of MSPs in order to keep the same experimental conditions as in our earlier study, conducted by Wigenstam et al. [35]. The optimal nebulizer concentration of DEX was determined from that study, and was set to be 5 mg/mL (DEX/HEPES). Consequently, in the present study, this concentration of free DEX was used as a control. The MSPs, comprising silica-DEX to a ratio of 50:50, were also administered as a wet aerosol in HEPES. The DEX-loaded L-MSPs were administered in two different concentrations—10 mg/mL (which corresponds to 5 mg/mL free DEX), and 5 mg/mL (corresponds to 2.5 mg/mL free DEX). As controls, empty L-MSPs were used in the concentrations of 5 mg/ml and 2.5 mg/mL, respectively, to keep the number of L-MSPs on the same level as the DEX-loaded L-MSPs. For the S-MSPs, only the lower concentration was chosen—5 mg/mL DEX-loaded S-MSPs and 2.5 mg/mL empty S-MSPs. Both the drug-loaded and the empty MSPs were surface-coated with the PEG–PEI copolymer.

A general drawback of the aerosol treatment is the difficulty to determine the exact internal drug dose from the nebulizer. In addition, the impact on the breathing pattern by the inflammatory response may lead to differences in the lung deposition of aerosol droplets between exposed and unexposed animals. For solid carrier particles, the final formulation would instead be devised as dried powder administered with the aid of dry powder inhalers (DPI). Nevertheless, it has recently been reported that the doses reaching the target tissue when administered from DPIs are in general far less (up to 10x) than the label claims [36], whereby a distinctly sized and shaped carrier particle able to rapidly release its payload would be crucial for predictable and successful therapy.

**Figure 2:**
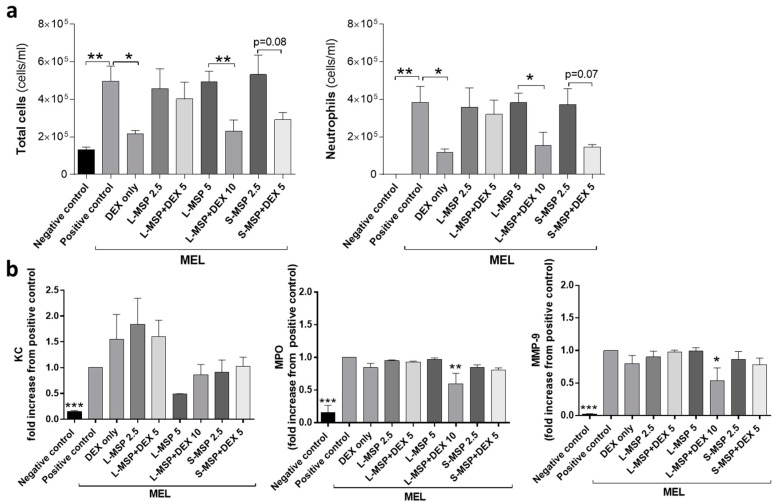
(**a**) Total leukocytes and the total number of neutrophils in bronchoalveolar fluid (BALF) and (**b**) analysis of (keratinocyte chemoattractant (KC), Mouse Myeloperoxidase (MPO), and Matrix metalloproteinase-9 (MMP-9)) in BALF from mice exposed to melphalan (MEL). Values indicate means ± SEM, * *p* < 0.05, ** *p* < 0.01, *** *p* < 0.001 compared to control groups. MSP—mesoporous silica particle (L—large; S—small), DEX—dexamethasone. Positive control: HEPES buffer administered to mice exposed to MEL/LPS; negative control: HEPES buffer administered to healthy mice; DEX only: 5 mg/mL (free DEX/HEPES buffer). The numbers on the x-axis denote mg/mL (particles/HEPES buffer). Each bar represents a group of *n* = 6 animals.

Both MEL- and LPS-exposure cause a neutrophilic inflammatory response in the lungs, and in the progression of the neutrophilic inflammatory response, MPO, KC, and MMP-9 are indicators of the activation and recruitment of neutrophils to the airways [37,38,39]. The results presented in Figure 2a reveal that the highest concentration of L-MSP loaded with DEX (L-MSP+DEX 10) provided a similar treatment effect to the MEL-induced lung inflammation, as a single aerosol of free DEX (5 mg/mL, “DEX only”). This was indicated by the reduced influx of neutrophils 24 h post exposure. Furthermore, the S-MSPs (S-MSP + DEX 5 mg/mL) reduced the cellular inflammation (*p* = 0.08) and neutrophils (*p* = 0.07) almost to the same degree, even though the administered amount of these particles carried only half of the amount DEX (2.5 mg/mL) compared to free DEX (5 mg/mL). As control groups, the HEPES buffer was administered either to the mice exposed to MEL/LPS (positive control), or to the healthy mice (negative control).

An analysis of the pro-inflammatory mediators in BALF (Figure 2b) revealed that the MSPs, in some cases, could decrease the level of MPO and MMP-9, while the free DEX had no effect on the mediator levels. The potential of nanoparticles (NPs) to bind proinflammatory cytokines has been previously demonstrated under in vitro conditions [40,41,42]. The ability of NPs to form complexes with proteins, leading to a protein corona [43,44] is a well-known phenomenon in NP drug delivery.

**Figure 3:**
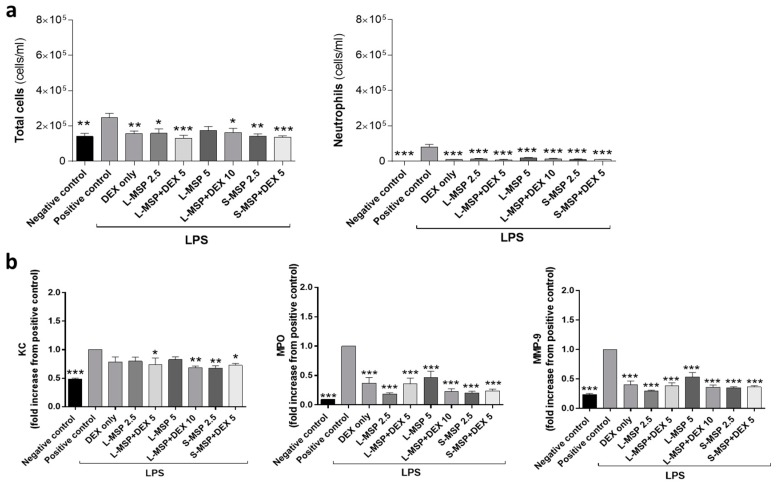
(**a**) Total leukocytes and the total number of neutrophils in bronchoalveolar fluid (BALF) and (**b**) analysis of KC, MPO, and MMP-9 in the BALF from the mice exposed to lipopolysaccharide (LPS). Values indicate means ± SEM; * *p* < 0.05, ** *p* < 0.01, *** *p* < 0.001 compared to the LPS group receiving vehicle. MSP—mesoporous silica particle (L: large, S: small); DEX—dexamethasone. Positive control: HEPES buffer administered to the mice exposed to MEL/LPS; negative control: HEPES buffer administered to healthy mice; DEX only: 5 mg/mL (free DEX/HEPES buffer). The numbers on the x-axis denote mg/mL (particles/HEPES buffer). Each bar represents a group of *n* = 6 animals.

Interestingly, in the LPS-model, the inflammatory response was significantly reduced in all of the MSP-treated groups. Regardless of the DEX-loading in the MSPs or not, all of the treated animals showed a reduced number of total cells and neutrophils 24 h after exposure, compared with the positive control group, as seen in Figure 3a. The concentrations of the pro-inflammatory mediators MPO and MMP-9 in the BALF were also significantly reduced in all of the treated groups (Figure 3b). The apparent down-modulatory effect of MSPs on the LPS-induced airway inflammation could hypothetically be explained by the different magnitudes of inflammation in the two mouse models. The extent of the MEL-induced inflammation was more elevated than the LPS-induced inflammation, which may be one possible explanation for the different results observed between the two models. High-dose exposure to MEL leading to tissue damage in both bronchial epithelium and endothelium, and induces a sustained pro-fibrotic inflammatory response in the lung [29,30]. Inhaled LPS induces an oxidative burst that can lead to excessive inflammation, which may lead to tissue injury. In this study, the inflammatory response of LPS was not excessive in comparison to the MEL group, and could possibly explain why the MSPs had little effect on the pro-inflammatory mediators in the MEL-model in comparison to the LPS group. As a result of the lower degree of inflammation in the LPS-model, the mediators may be transported or carried easier by the MSPs to clear the BALF, thereby also influencing the inflammatory response [38]. The lack of correlation between the pro-inflammatory mediators and the reduced levels of neutrophils could be due to timing, as different time frames were used when administering aerosolized DEX to get a maximum anti-inflammatory effect. There is also a possibility that the DEX encapsulated in the MSPs may delay the release of DEX, making a similar dose of DEX (L-MSP + DEX 10 mg/mL) more anti-inflammatory than one dose of free DEX. To our understanding, the previous studies that have used and administered corticosteroid treatment as an aerosol have administered the drug at several time-points or at a longer duration than the experimental set-up in this study.

Overall, the potential of the drug-loaded MSPs, both S-MSPs and L-MSPs, on the treatment of airway inflammation seems promising. The MSPs did not cause any visible toxic effects, and there were no differences in behavior observed between the exposed and non-exposed animals.

## 4. Conclusions

In this study, mesoporous silica particles (MSPs) of two different sizes have been produced and characterized, and further evaluated as carriers for the corticosteroid dexamethasone (DEX). Their feasibility as delivery vehicles for treating airway inflammations in mice, which in the present case was induced by melphalan (MEL) or lipopolysaccharide (LPS), was investigated. The results showed that the MEL-induced neutrophilic airway inflammation could be treated by aerosolized MSP-encapsulated DEX, at least to the same extent as free DEX. Interestingly, in the MEL-induced inflammation model, inhaled empty particles had no apparent effect on inflammation; while, in the LPS-induced inflammation model, inhaled particles significantly down-modulated the inflammatory response regardless of the presence of DEX or not. The results from the MEL-exposed mice indicate that MSPs are viable drug carriers for pulmonary delivery of corticosteroids in the anti-inflammatory treatment of chemical-induced lung injury. The mechanism by which empty MSPs reduced the LPS-induced airway inflammation is presently not known, but should be the focus of future studies. Hypothetically, this observation could be explained by different magnitudes of inflammation in the two models, and, potentially, also by interactions of MSPs with proteins and cells in BALF. By using mouse models for the in vivo treatment of toxic chemical- and endotoxin-induced lung injury by MSPs loaded with corticosteroids, we have shown that inhaled MSPs can exert therapeutic action in inflammatory conditions. The results presented here provide a foundation for future studies aimed at identifying new concepts for treatment using MSP-based drug delivery.

## Figures and Tables

**Table 1:** Groups and experimental protocols.

Exposure	Particle Size	Group	Particle Dose/Vehicle	DEX Dose
Solvent		Control	HEPES	-
MEL or LPS exposure		MEL or LPS	HEPES	-
		DEX		5 mg/mL
	L-MSP (1 µm)	L-MSP 2.5	2.5 mg/mL	-
	L-MSP (1 µm)	L-MSP+DEX 5	5 mg/mL	2.5 mg/mL
	L-MSP (1 µm)	L-MSP 5	5 mg/mL	-
	L-MSP (1 µm)	L-MSP+DEX 10	10 mg/mL	5 mg/mL
	S-MSP (200 nm)	S-MSP 2.5	2.5 mg/mL	-
	S-MSP (200 nm)	S-MSP+DEX 5	5 mg/mL	2.5 mg/mL

MEL—melphalan; LPS—lipopolysaccharide; MSP—mesoporous silica nanoparticle; DEX—dexamethasone; vehicle—HEPES buffer (25 mM, pH 7.2); L-MSP—large MSP; S-MSP—small MSP.

## Supplementary Materials

The following are available online at https://www.mdpi.com/1999-4923/11/4/149/s1: Figure S1. Five different extraction methods for removing the hexadecylamine surfactant template were compared; Figure S2. The particles extracted with acidic ethanol have higher specific surface area probably due to more efficient removal of the hexadecylamine surfactant from the pore structure; Figure S3. Thermogravimetric measurements confirm that residues of the surfactant were most likely still present in the pores after extraction with isopropanol; Figure S4. Release of dexamethasone from the silica particles coated with PEG-PEI copolymer.
