# Supplementary material for: Feasibility Study of Mesoporous Silica Particles for Pulmonary Drug Delivery: Therapeutic Treatment with Dexamethasone in a Mouse Model of Airway Inflammation
Source: Pharmaceutics. 2019 Apr 1;11(4):149. doi: 10.3390/pharmaceutics11040149 (PMC6523761; doi:10.3390/pharmaceutics11040149)
Supplement: Supplementary file 1 [file pharmaceutics-11-00149-s001.pdf]

Supplementary data to: Feasibility study of mesoporous silica particles for pulmonary drug delivery: therapeutic treatment with dexamethasone in a mouse model of airway inflammation

Tina Gulin-Sarfraz, Sofia Jonasson, Elisabeth Wigenstam, Eva von Haartman, Anders Bucht*, Jessica M. Rosenholm*

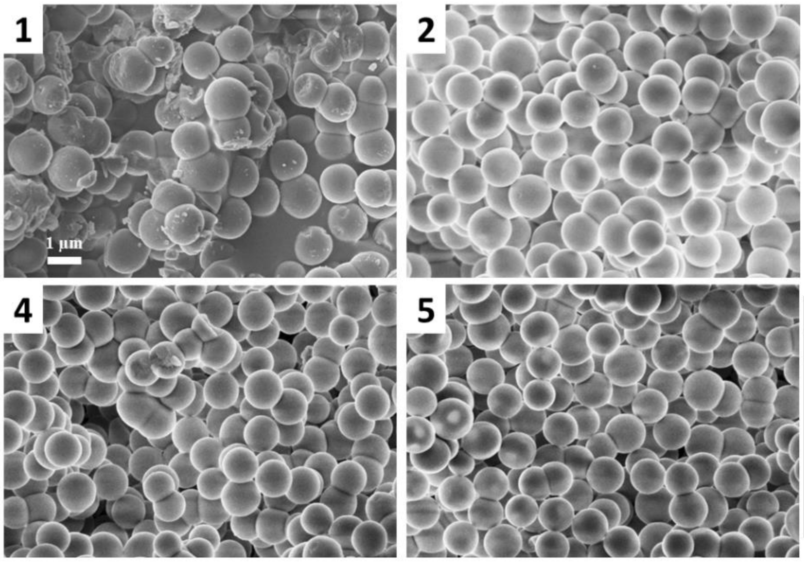

Sample	Extraction method
1	Ethanol + high acid + sonication
2	Isopropanol + sonication
3	Ethanol + low acid + sonication
4	Isopropanol
5	Ethanol + low acid

Supplementary Fig. S1 Five different extraction methods for removing the hexadecylamine surfactant template were compared. The extraction methods are listed in the table. Acidic ethanol (high acid = 1.3 M HCl, low acid = 0.1 M HCl) or isopropanol were used as extraction solvents together with either powerful sonication treatment or gentle stirring/shaking. Scanning electron microscopy (SEM) images reveal that acidic ethanol together with sonication (sample 1 and 3) damaged the particle structures and consequently also caused aggregation of the particles. When extracting with only isopropanol the particle structures were kept intact independent of sonication treatment (sample 2 and 4). Careful extraction with acidic ethanol (sample 5) did not either negatively affect the particles. Since both of the differently sized particles used for the study, L-MSP and S-MSP, are synthesized according to the same synthesis strategy, only the larger particles were chosen as models for evaluating the different extraction methods.

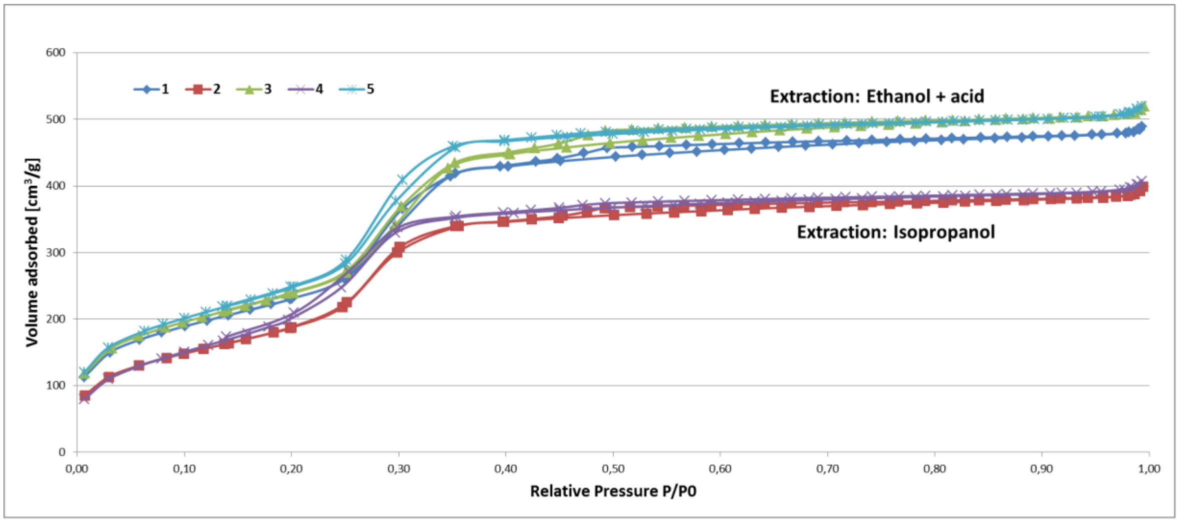

Sample	Extraction method	BET [m^2/g]	Pore size [nm]	Pore volume [cm^3/g]
1	Ethanol + high acid + sonication	846	3.7	0.71
2	Isopropanol + sonication	699	3.7	0.57
3	Ethanol + low acid + sonication	875	3.8	0.75
4	Isopropanol	767	3.5	0.58
5	Ethanol + low acid	912	3.7	0.75

Supplementary Fig. S2 The particles extracted with acidic ethanol have higher specific surface area probably due to more efficient removal of the hexadecylamine surfactant from the pore structure. The particles extracted with isopropanol have clearly lower BET surface area and pore volume, independent of sonication treatment during the extraction. These nitrogen sorption isotherms, together with the SEM images in Fig. S1, thus reveal that the extraction method number 5 in the table (0.1 M HCl in ethanol, without sonication) is the best SDA removal method for the particles synthesized according to the here presented synthesis route.

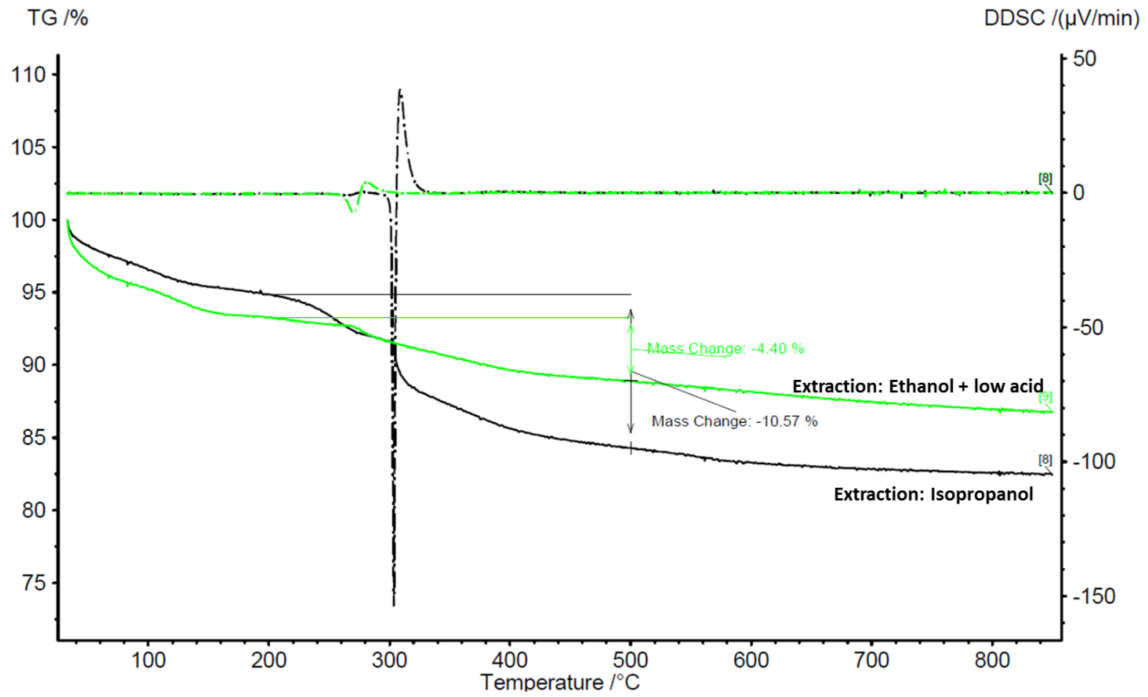

Supplementary Fig. S3 Thermogravimetric measurements confirm that residues of the surfactant were most likely still present in the pores after extraction with isopropanol. The hexadecylamine surfactant burns around 300 °C, which can be seen in the measurement of the particles extracted with isopropanol, as a mass loss of about 6 %. The "Ethanol + low acid" extracted sample show a TGA curve typical for amorphous silica.

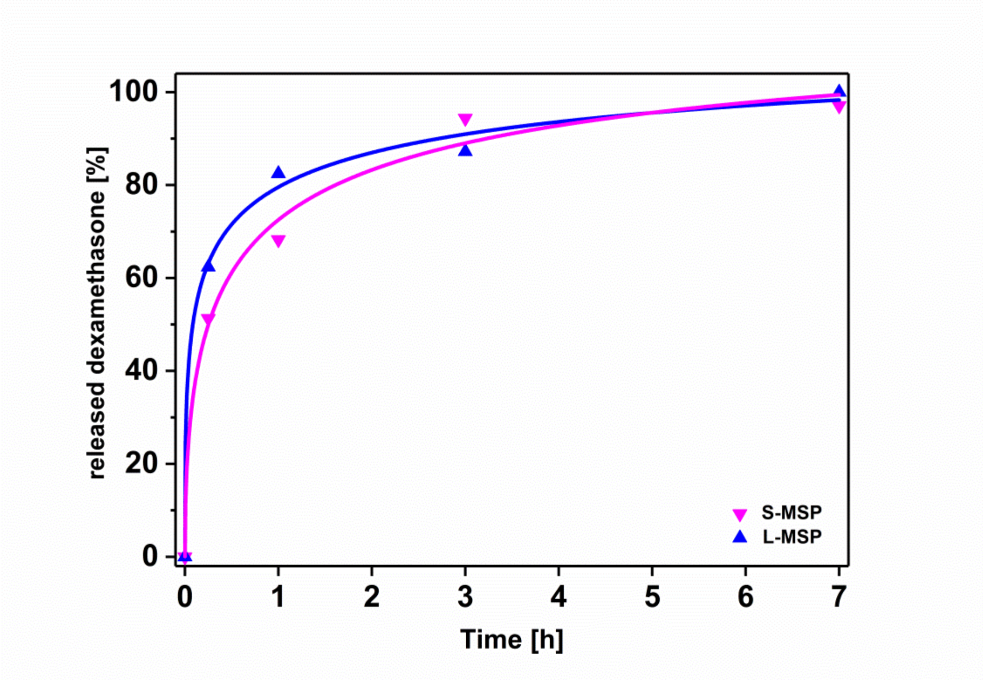

Supplementary Fig. S4 Release of dexamethasone from the silica particles coated with PEG-PEI copolymer.
